# Case report: A case of pulmonary mucormycosis caused by *Rhizopus azygosporus* infection complicated by type 2 diabetes mellitus

**DOI:** 10.3389/fmed.2023.1240436

**Published:** 2023-10-06

**Authors:** Lu Wang, Yuanqing Qu, Lu Tang, Yanmei Li, Lu Liu, Yuan Liu

**Affiliations:** Department of Laboratory Medicine, PLA Western Theater Command General Hospital, Chengdu, China

**Keywords:** type 2 diabetes, *Rhizopus azygosporus*, mucormycosis, pneumonia, case

## Abstract

A case of pulmonary mucormycosis (PM) caused by *Rhizopus azygosporus* infection complicated by type 2 diabetes mellitus is reported. An adult male patient had a productive cough for more than 10 days, aggravated by blood in the sputum for 9 days. Laboratory examination confirmed that he had had type 2 diabetes mellitus and diabetic ketosis for more than 3 years, and his chest computed tomography (CT) scan showed lesions, cavities, and a small effusion in the right lower lobe. The lavage fluid was taken by bronchoscope for bacterial culture and mNGS, which indicated *Rhizopus azygosporus* growth. Mucormycosis was diagnosed. The patient was given amphotericin B cholesterol sulfate complex for 30 days, and his renal function was closely monitored. After that, his right lower lobe was resected. To date, the patient has recovered well.

## 1. Introduction

Mucormycosis is a life-threatening fungal infection caused by molds, known as mucormycetes (1). It usually affects patients with serious underlying conditions, such as poorly controlled diabetes (e.g., ketoacidosis or hyperosmolar coma), hematological malignancies, or those undergoing hematopoietic stem cell transplantation, glucocorticoid and/or immunosuppressive therapy, solid organ transplantation, iron overload, severe influenza, acquired immunodeficiency syndrome (AIDS), burns or other traumatic injuries, and severe malnutrition (2–5). Common sites of infection are the sinuses, lungs, skin, brain, and gastrointestinal tract. Poorly controlled diabetes has long been recognized as an important risk factor for mucormycosis. The etiologic diagnostic approaches to mucormycosis involve microbiology (6, 7), histopathology (8), and molecular biology (9). Host factors, clinical manifestations, and microbiological evidence are indispensable for the clinical diagnosis of mucormycosis. The treatment of mucormycosis should first involve actively controlling the underlying diseases. An important principle of the treatment is to carry out early surgical procedures whenever possible, including local debridement and resection of infected tissues or organs (10, 11). Systemic antifungal therapy is also necessary for mucormycosis, including the use of amphotericin B lipid preparations and AmB deoxycholate, posaconazole, and isavuconazole (12, 13). Currently, there are few reports of invasive mucormycosis in patients with type 2 diabetes. Here we present a rare case of pulmonary mucormycosis caused by type 2 diabetes complicated by *Rhizopus azygosporus* infection.

## 2. Case presentation

On 17/04/2023, an adult male patient presented to the Department of Respiratory Infections due to a productive cough of more than 10 days’ duration. The cough was aggravated by the presence of blood in the sputum for 9 days. The patient had previously been diagnosed with type 2 diabetes mellitus for more than 3 years, which was poorly managed with inadequate blood sugar control. On 7/04/2023, he underwent a chest computed tomography (CT) scan at another hospital, which revealed a right lower lobe mass. Considering the possibility of infectious lesions, cephalosporin antibiotics were then used (details unknown), but there was no improvement. Therefore, he came to our hospital for treatment and was admitted for “suspected lung abscess and type 2 diabetes mellitus with ketoacidosis.”

**Figure 1:**
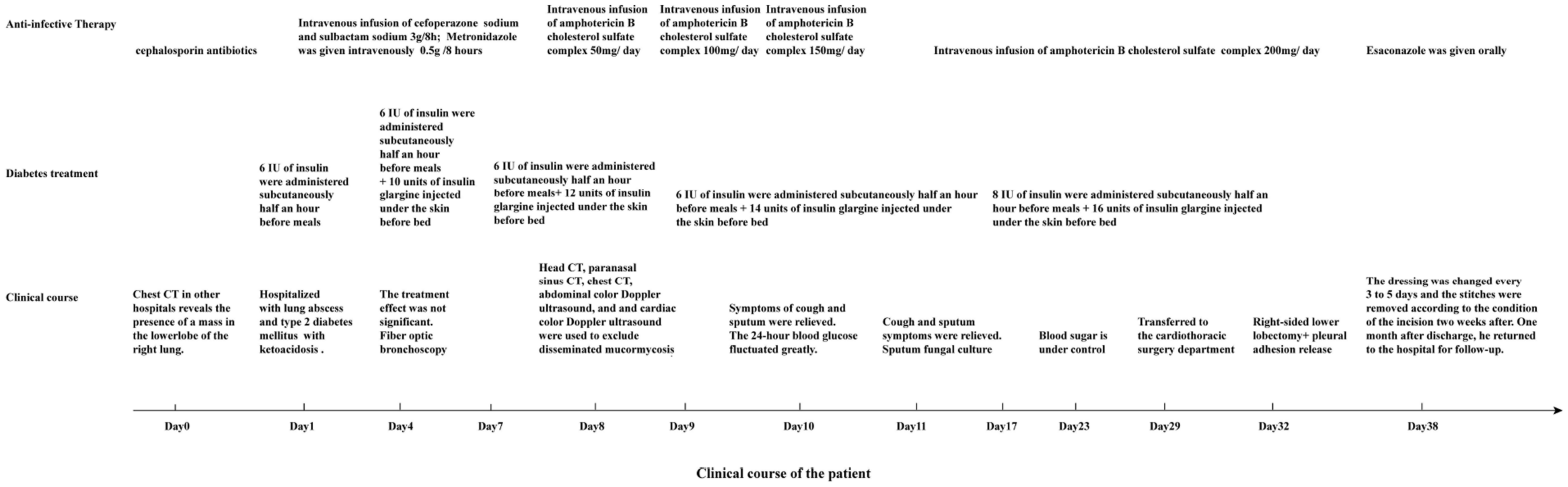
The clinical progression of the patient’s condition and the treatments received.

Laboratory tests and auxiliary examinations were conducted on the patient (Table 1). His electrocardiogram showed sinus rhythm with a heart rate of 91 beats/min, and chest CT revealed a large mass shadow with a cavity in the right lower lobe. A thick-walled cavity with a large cross-sectional area of about 7.6 cm*7.9 cm was observed, with its internal septa and gas–liquid level formed and surrounded by many fuzzy patches and strips. The interlobular septa were partially thickened. There was a small amount of bilateral effusion in the chest cavity. Following admission due to a pulmonary infection, the patient received symptomatic and supportive care, which included antibiotic therapy with a combination of cefoperazone/sulbactam sodium and metronidazole, antihypertensive therapy with insulin administration, treatment of electrolyte imbalance, rehydration, and oxygen inhalation. Since the patient’s blood sugar was poorly controlled, he was advised to follow a diabetic diet. For insulin therapy, he received a subcutaneous injection of 6 IU of insulin before meals and an injection of 10 units of insulin glargine before going to bed. The therapies used to regulate glucose are shown in Figure 1.

**Table 1:** Laboratory tests and auxiliary examinations.

Examination		Result	Reference values
Routine blood tests	WBC	12.39*10^9^/L	3.50–9.50*10^9^/L
	NEUT%	78.2%	40.0–75.0%
	LYM%	13.8%	20.0–50.0%
	EOS%	2.1%	0.4–8.0%
	RBC	4.26*10^12^/L	4.30–5.80*10^12^/L
	HGB	121 g/L	130–175 g/L
	PLT	464*10^9^/L	125–350*10^9^/L
	hs-CRP	40.11 mg/L	0–3 mg/L
	ESR	34.00 mm/h	0–21 mm/h
Coagulation function	D-Dimer	0.70 mg/L(FEU)	0–0.55 mg/L(FEU)
	FIB	7.23 g/L	2.00–4.00 g/L
Liver function	ChE	4.5*10^3^U/L	5.1–11.7*10^3^U/L
	PA	93 mg/L	180–390 mg/L
	Alb	29.2 g/L	40.0–55.0 g/L
	TP	59.4 g/L	65.0–85.0 g/L
	A/G	0.97	1.20–2.40
Renal function	Urea	2.04 mmol/L	2.90–7.20 mmol/L
Electrolytes	Ca^2+^	1.94 mmol/L	2.00–2.70 mmol/L
	CI^−^	94.6 mmol/L	99.0–110.0 mmol/L
	Na^+^	127.3 mmol/L	137.0–147.0 mmol/L
Urine routine test	Glucose in urine	4+	Negative
Measurement of glucose	Random blood sugar	20.01 mmol/L	3.80–6.10 mmol/L
*Mycoplasma pneumoniae* antibody titer	1:160	+	Negative
Cytokines	IL-6	41.56 pg/mL	0–5.30 pg/mL
Tumor marker			
Lung cancer	SCC	1.02 ng/mL	0–2.70 ng/mL
	NSE	13.58 ng/mL	0–17.00 ng/mL
	CYFRA21-1	1.97 ng/mL	0–3.30 ng/mL
Galactomannan (GM) test			
TB	T-cellγinterferon release assay	Negative	Negative
	Tuberculosis antibody test	Negative	Negative
	*Mycobacterium tuberculosis* DNA fluorescence quantitative PCR	Negative	Negative

**Figure 2:**
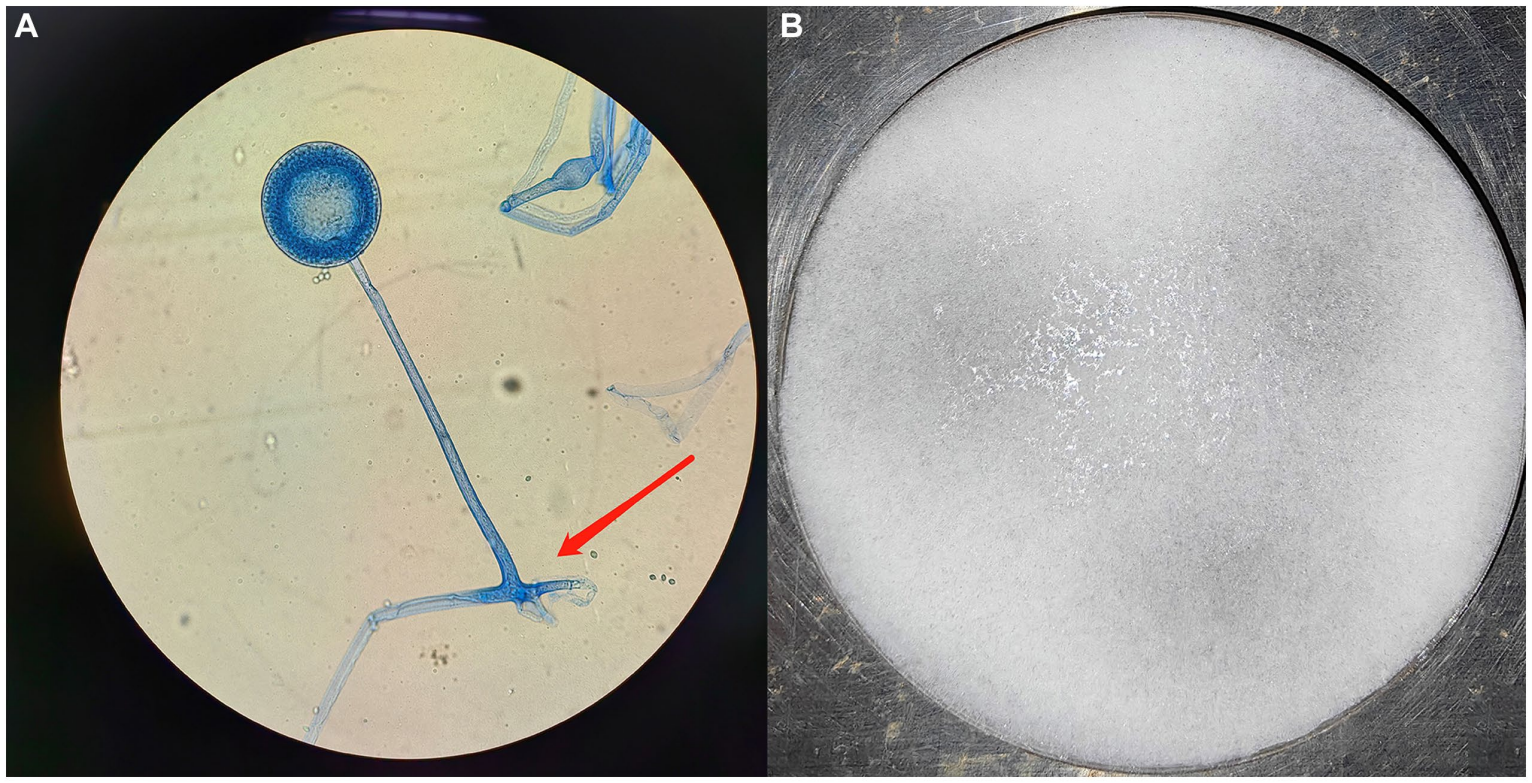
**(A)** Microscopic examination of the patient’s Rhizopus: PDA, cultured at 28°C for 3 days, stained with lactophenol cotton blue; broad, non-septate, subrectangular branching hyphae, suggestive of Trichoderma mycelium, were observed, x100; **(B)** Rhizopus colony in the patient: FDA, cultured at 28°C for 3 days.

**Figure 3:**
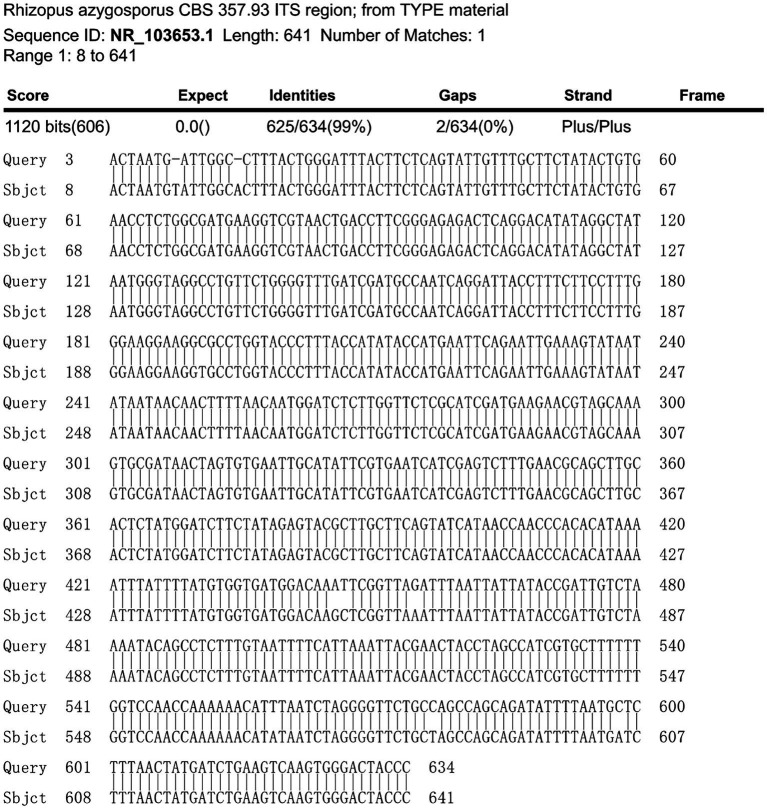
The results of PCR sequencing.

**Figure 4:**
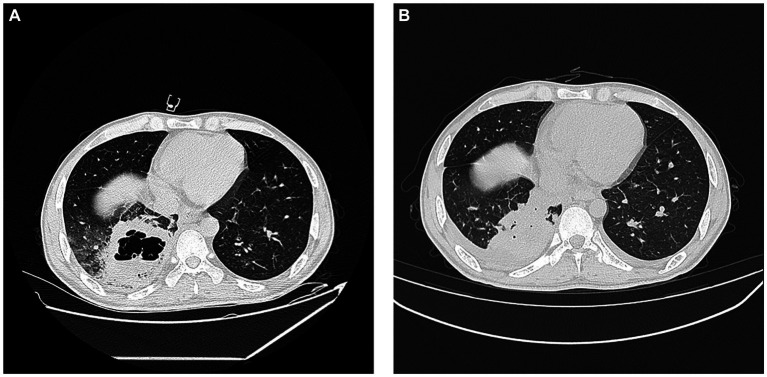
Lung conditions upon admission and after treatment. **(A)** Chest CT on initial admission; **(B)** 17th day of hospitalisation chest CT. Pulmonary fuzzy spots and patches and high density in parts of the lungs, which were slightly absorbed and thinner than before. The cavity in the right lower lobe is smaller than before, the gas shadow is reduced, and the liquid density is increased.

A fiber optic bronchoscopy was performed on the patient on Day 4 of admission, which revealed a cavity in the distal right lower lobe posterior bronchus. The lavage fluid sent for metagenomic next-generation sequencing (mNGS) suggested *Rhizopus azygosporus*. In addition, LPCB staining was performed for microscopy (Figure 2A). The fungal culture of the lavage fluid showed *Rhizopus* growth (Figure 2B). Pure fungal colonies were sent for detection, and the internal transcribed spacer (ITS) sequence was amplified by polymerase chain reaction (PCR). The colonies were finally identified as *Rhizopus azygosporus* (Figure 3). The patient was diagnosed with an invasive fungal disease (mucormycosis). Antifungal susceptibility testing of filamentous fungi showed that the strain was susceptible to amphotericin B. The patient was then treated with systemic antifungal drugs according to the 2022 Chinese Expert Consensus on Clinical Diagnosis and Treatment of Mucormycosis. Based on the results of relevant fungal susceptibility tests from the Department of Basic Medical Laboratory, the antifungal treatment was initiated on Day 8 post-admission. Intravenous amphotericin B cholesterol sulfate 50 mg/day was initially administered, and then the dose was gradually increased to 200 mg/day. The efficacy of the treatment was assessed according to the patient’s symptoms and the results of the chest CT. On the 17th day of hospitalization, the chest CT scan showed a reduction in cavity size in the lower lobe of the right lung, with a decrease in air density and an increase in fluid density. The result indicated an improvement in the lung lesion compared to the initial CT scan conducted at admission. Figures 4A,B show the lung conditions on admission and after treatment. The patient’s condition was evaluated in consultation with doctors from the Cardiothoracic Surgery Department, and the re-examined fiberoptic bronchoscopy showed that there was no obvious abnormality in the trachea or bronchus. The pulmonary function test suggested that the lung ventilation function was slightly impaired; the airflow in the large airway was slightly restricted, and the airflow in the small airway was slightly moderately obstructed. Diffusion function was slightly decreased, and the RV/TLC was normal. After taking amphotericin B for 21 days, the patient’s condition improved significantly compared with that on admission. He was then transferred to the Cardiothoracic Surgery Department on Day 29 of his hospitalization. In order to remove the pulmonary lesion, clarify the nature of the lesion, prolong survival time, and improve the quality of life of the patient, lobectomy of the right lower lobe and thoracolysis were performed on the 32nd day of hospitalization. Surgery revealed that the lesion was located in the lower lobe parenchyma of the right lung and was intimately attached to the diaphragm and chest wall, measuring approximately 5.0 cm*5.0 cm*3.0 cm, with a significant amount of yellowish-black necrotic tissue visible on sectioning. The excised tissue was subjected to pathological special staining, periodic acid-Schiff’s staining, and acid-fast staining, all of which were negative. Antifungal therapy with amphotericin B was continued after surgery. The patient recovered well after surgery. He was discharged from the hospital to continue anti-infection treatment with oral isavuconazole; the dressing for the surgical incision was changed after 3–5 days; the stitches were removed according to the condition of the incision, and the patient returned to the hospital for follow-up after one month. The clinical progression of the patient’s condition and the treatments received are shown in Figure 1.

## 3. Discussion

PM is a rare and fatal pulmonary infection caused by Mucorales fungi, and its incidence rate is increasing every year. It mainly occurs in patients with hematological malignancies who have undergone hematopoietic stem cell transplantation and often affects those with long-term neutropenia or during the treatment of severe graft-versus-host disease (14, 15), followed by those with diabetic ketoacidosis (16). Especially in recent years, with the global outbreak of the COVID-19 pandemic, the pulmonary diseases caused by mucormycosis have rapidly progressed, resulting in a worse prognosis, with a mortality rate exceeding 57% (17) and a case fatality rate close to 40% in China (18).

The clinical and imaging manifestations of PM lack specificity; therefore, it is difficult to diagnose the pathogenic bacteria quickly and accurately. The susceptible populations, high-risk factors, CT manifestations, and dynamic evolution process of PM are similar to those of Aspergillus infection, which easily leads to misdiagnosis in clinical settings (19). In terms of traditional laboratory detection methods, the etiology of PM can be determined by methods related to microbiology, histopathology, and molecular biology (20). In high-risk patients, microbiological and histopathological examinations should be actively carried out. Traditional microbiological detection is time-consuming, and it is difficult to identify specific species. Gram staining, potassium hydroxide (KOH) smears, immunofluorescence staining, or methenamine silver staining allow direct microscopic examination of fungi. Sputum or bronchoalveolar lavage fluid (BALF) specimens can be directly smeared. If Mucorales hyphae are found, a Mucor infection is highly suspected. In addition, qualified fungal specimens from the lower respiratory tract can also be cultured. Mucor culture usually takes about 3–5 days. After this time, the fungal hyphae are well developed and fill the entire culture dish, initially with white wool-like colonies, then turning gray. Commonly used fungal antigens (such as (1,3)-β-D glucan detection and galactomannan detection) have low sensitivity and specificity for Mucor (21). As a new technology for pathogen detection, metagenomic next-generation sequencing (mNGS) has been gradually popularized in the field of fungal detection in recent years. Due to its characteristics of unbiased, broad coverage, high sensitivity, and high speed (22), it is relatively quick to diagnose rare Mucor infections and their mixed infections when combined with laboratory detection. Especially by identifying the pathogens to the strain level by mNGS, this plays a guiding role in timely clinical medication (9, 23). After 3 days of anti-infection treatment for the patient admitted to our hospital showed no effect, samples were taken for mNGS and bacterial culture by fiberoptic bronchoscope. The next day, the results revealed Rhizopus azygosporus. After 3 days, the results of microbial culture and microscopic examination were consistent with the growth pattern of Rhizopus, which was very helpful for clinicians to adjust the antifungal treatment plan in time. The treatment of PM mainly includes antifungal drug treatment and surgical treatment. Amphotericin B is the first choice for antifungal treatment (24). This patient has had type 2 diabetes mellitus for many years. If he were to stop taking anti-infective drugs in the later recovery stage, the high blood sugar environment in his body would easily lead to another outbreak of infection. Therefore, the doctor decided to operate and completely remove the lung lesions to avoid future problems.

The clinical manifestations of diabetes mellitus complicated by PM are not typical, and the disease progresses rapidly with a poor prognosis and high mortality. Clinically, it is necessary to improve the understanding of rare complications of diabetes mellitus and consider the special cases of rare fungal infections. The clinical diagnosis of mucormycosis is based on host factors, clinical manifestations, and microbiological evidence (25). The emerging technology of mNGS, combined with traditional laboratory detection and imaging, is the key to quickly diagnosing pathogenic bacteria. For the special internal environment of diabetic patients, it is necessary to work out a careful plan for clinical antifungal treatment, monitor the patient’s condition, adjust the medication in time, and combine it with surgical treatment in later stages if appropriate.

## Data availability statement

The original contributions presented in the study are included in the article/Supplementary material, further inquiries can be directed to the corresponding author.

## Ethics statement

The studies involving humans were approved by the General Hospital of Western Theater Command of PLA. The studies were conducted in accordance with the local legislation and institutional requirements. The participants provided their written informed consent to participate in this study. Written informed consent was obtained from the individual(s) for the publication of any potentially identifiable images or data included in this article.

## Author contributions

LW: conceptualization and writing – review and editing. YQ: methodology and resources. YaL: formal analysis and investigation. LT: writing – original draft preparation. YuL: funding acquisition. LL: supervision. All authors contributed to the study conception and design, commented on earlier versions of the manuscript, read, and approved the final version of this work.
